# Supplementary material for: Highly efficient expression of DNA-peptide conjugates in growth-arrested cells
Source: Nat Commun. 2026 Jan 7;17:1422. doi: 10.1038/s41467-025-68167-5 (PMC12881617; doi:10.1038/s41467-025-68167-5)
Supplement: Supplementary file 1 — Supplementary Information [file 41467_2025_68167_MOESM1_ESM.pdf]

Supplementary Information

Supplementary Method 1. DNA Oligonucleotides and plasmid sequences

Oligonucleotide stem loops used in this work for DNA oligonucleotide-peptide conjugates and ligation to expression cassettes were ordered from Integrated DNA Technologies. DNA-TAG recognition stem loops are underlined; sticky end portion for ligation is bolded. Stem loops were modified on the 5′ position with a phosphate to facilitate ligation.

Supplementary Table 1. DNA stem loop sequences

Stem loop	Sequence
MluI 170 PP	5′-5/Phos/**CGCG**<u>CCGGATTGTCCTTCCGG</u>-3′
AvrII 170 PP	5′-5/Phos/**CTAG**<u>CCGGATTGTCCTTCCGG</u>-3′
AflIII 170 PP	5′-5/Phos/**CGCG**<u>CCGGATTGTCCTTCCGG</u>-3′

The *E.coli* Strep-TGT-His plasmid construct from which TGT was expressed and purified is found on Addgene (206495).[1]

All peptide modified gene cassettes used for transfection experiments are driven by the same CMV enhanced and promoter and have the same bGH poly(A) signal found within the pcDNA3.1 plasmid vector to keep the expression of genes following mammalian transfection similar.

The pcDNA3.1 (+) eGFP (Addgene 129020) plasmid was used for generation of the 2537 bp capped linear gene cassette for transfection. The 5′ MluI and 3′ AvrII restriction sites are underlined in the coding sequence provided in Supplementary Data 1.

The NLS-eGFP (Addgene 67652) plasmid was used for expression of NLS-eGFP in mammalian cells to determine the dose of importazole needed to reduce nuclear transport mediated by NLS-importin interactions.

A miniGFP1 (Addgene 188896) plasmid construct was generated by ordering a miniGFP1 gene block from Twist Biosciences.[2] The gene block was designed to have a 5′ MluI restriction site and a 3′ AvrII restriction site. The pcDNA3.1 (+) eGFP and miniGFP1 gene block were digested with these restriction enzymes from New England Biosciences, and the resulting sticky end DNA fragments were ligated to generate pcDNA3.1 miniGFP1. The resulting plasmid was verified by whole plasmid sequencing by Plasmidsaurus. The miniGF1 plasmid was used for the generation of the 1361 bp capped linear gene cassette for transfection. The 5′ MluI and 3′ AvrIII restriction sites are underlined in the coding sequence provided in Supplementary Data 1.

The ABE8e-P2A-eGFP plasmid was used for the generation of the 6979 bp capped linear gene cassettes for transfection.[3] The 5′ MluI and 3′ AflIII restriction sites are underlined in the coding sequence provided in Supplementary Data 1.

A human coagulation factor IX plasmid construct was generated by ordering a factor IX gene block from Twist Biosciences. The gene block was designed to have a 5′ MluI restriction site and a 3′ AvrII restriction site. The pcDNA3.1 (+) eGFP and factor IX gene block were digested with these restriction enzymes from New England Biosciences, and the resulting sticky end DNA fragments were ligated to generate pcDNA3.1

factor IX. The resulting plasmid was verified by whole plasmid sequencing by Plasmidsaurus. The factor IX plasmid was used for the generation of the 2539 bp capped linear gene cassette for transfection. The 5′ MluI and 3′ AvrII restriction sites are underlined in the coding sequence provided in Supplementary Data 1.

Supplementary Table 2. Peptides synthesized with a C terminal azido-modified lysine and flexible GGGGS linkers. NLS portions are highlighted in green; linker portions are highlighted in red. For the SV40 KO peptide, the mutated amino acid conferring NLS knock out is bolded and underlined.

Peptide	mw	Sequence (N→C)
SV40 NLS Lx1	1944.16	PKKKRKVEDPYS$(GGGGS)_1K(N_3)$
SV40 NLS Lx2	2259.45	PKKKRKVEDPYS$(GGGGS)_2K(N_3)$
SV40 NLS Lx3	2574.73	PKKKRKVEDPYS$(GGGGS)_3K(N_3)$
SV40 NLS Lx4	2890.02	PKKKRKVEDPYS$(GGGGS)_4K(N_3)$
SV40 NLS Lx5	3205.30	PKKKRKVEDPYS$(GGGGS)_5K(N_3)$
Influenza A Virus A NP NLS Lx1	2976.10	MASQGTKRSSYEQMETDGERQS$(GGGGS)_1K(N_3)$
Influenza A Virus A NP NLS Lx2	3291.39	MASQGTKRSSYEQMETDGERQS$(GGGGS)_2K(N_3)$
Influenza A Virus A NP NLS Lx3	3606.68	MASQGTKRSSYEQMETDGERQS$(GGGGS)_3K(N_3)$
SV40 KO Lx3	2547.66	PK**T**KRKVEDPYS$(GGGGS)_3$K(N3)
extSV40 NLS Lx3	4031.13	SSDEEATADQHSTPPKKKRKVEDPYS$(GGGGS)_3K(N_3)$
Borna disease virus p10 (BVP) NLS Lx3	2936.21	LRLTLLELVRRLNGNG$(GGGGS)_3K(N_3)$
HTLV-1 NLS Lx3	3404.77	MPKTRRRPRRSQRKRPPT$(GGGGS)_3K(N_3)$
IGFBP2 NLS Lx3	3203.51	KHHLGLEEPKKLRPPPAR$(GGGGS)_3K(N_3)$
Hrp-1 NLS Lx3	4186.27	RSGGNHRRNGRGGRGGYNRRNNGYHPY$(GGGGS)_3K(N_3)$
PLSCR-1 NLS Lx3	2226.34	GKISKHWTGI$(GGGGS)_3K(N_3)$

Supplementary Figure 1. Urea-PAGE of DNA oligonucleotide - peptide conjugates.

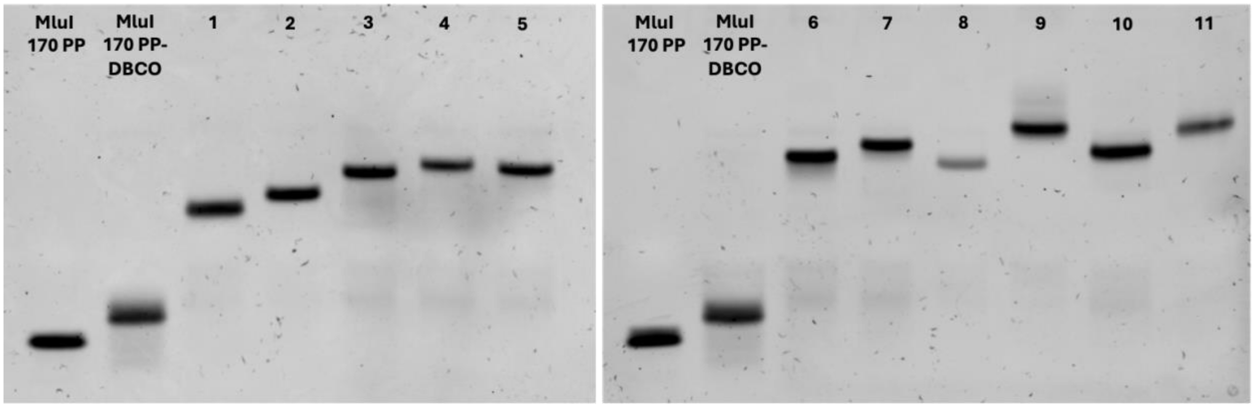

Urea-PAGE analysis of TGT-mediated labeling of MluI 170 PP DNA oligonucleotide with preQ$_1$-DBCO. Upward gel shift indicates successful covalent labeling. Lanes 1 – 11 depict the formation of various DNA oligonucleotide-peptide conjugates via strain-promoted azide-alkyne cycloaddition with near quantitative yields. Lane 1 – SV40 NLS Lx1, Lane 2 – SV40 NLS Lx2, Lane 3 – SV40 NLS Lx4, Lane 4 – SV40 NLS Lx5, Lane 5 – Influenza NLS Lx1, Lane 6 – Influenza NLS Lx2, Lane 7 – Influenza NLS Lx3, Lane 8 – BVP NLS Lx3, Lane 9 – HTLV-1 NLS Lx3, Lane 10 – IGFBP2 NLS Lx3, Lane 11 – Hrp-1 NLS Lx3. Gels are representatives of 3 independent experiments.

Supplementary Figure 2. Urea-PAGE of DNA oligonucleotide – peptide conjugates with different staining dyes.

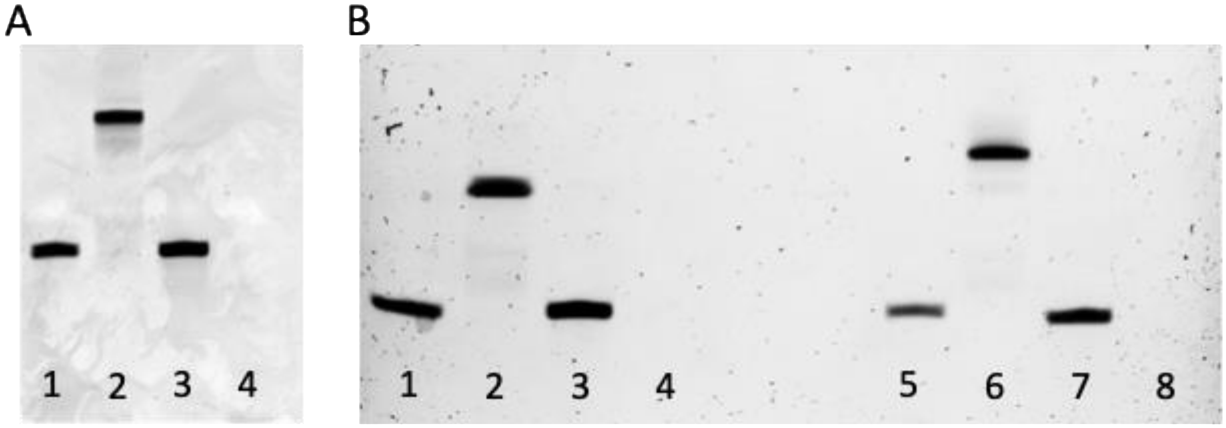

Urea-PAGE analysis of TGT-mediated labeling of MluI 170 PP DNA oligonucleotide with preQ$_1$-DBCO and peptides. Upward gel shift indicates successful covalent labeling. (A) depicts a gel stained with GelRed. Lane 1 – MluI 170 PP-DBCO, Lane 2 – MluI 170 PP-extSV40, Lane 3 - MluI 170 PP + extSV40 (not conjugated), and Lane 4 – extSV40. (B) depicts a gel stained with SYBER Safe. Lane 1 – MluI 170 PP-DBCO, Lane 2 – MluI 170 PP-extSV40, Lane 3 - MluI 170 PP + extSV40 (not conjugated), Lane 4 – extSV40, Lane 5 - MluI 170 PP-DBCO, Lane 6 - MluI 170 PP-PLSCR-1, Lane 7 - MluI 170 PP + PLSCR-1 (not conjugated), and Lane 8 – PLSCR-1. Gels are representative of 3 independent experiments

Supplementary Figure 3. Example agarose gel electrophoresis showing the generation of NLS modified capped linear eGFP gene cassettes.

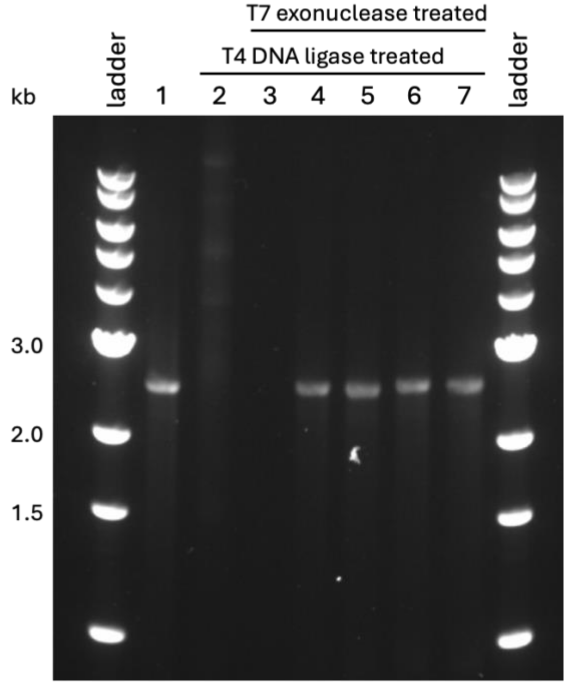

Lane 1 shows the AvrII and MluI-HF digested eGFP PCR product, generating a sticky ended eGFP gene fragment for ligation. Lanes 2 – 7 display T4 DNA ligation products, incubated either without (lanes 2–3) or with (lanes 4–7) 5′-phosphorylated DNA oligonucleotide stem loops corresponding to the sticky end eGFP gene fragment. Lane 3 – 7 show the DNA ligation products following T7 exonuclease treatment to ensure fully ligated, capped linear gene cassettes. Bands observed in lane 2 likely represent self-ligation products that were substrates for exonuclease treatment (3). Lanes 4 – 7 demonstrate the successful generation of capped linear eGFP gene cassettes, where the AvrII 170 PP DNA oligonucleotide stem loop was used to cap the 3′ end. The 5′ end was capped using: MluI 170 PP (4), MluI 170 PP – SV40 KO Lx3 (5), MluI 170 PP – SV40 NLS Lx3 (6), and MluI 170 PP – Influenza NLS Lx3 (7). A similar strategy was employed to generate capped linear gene cassettes for miniGFP1, ABE8e-P2A-eGFP, and factor IX, with or without NLS modifications. The gel is representative of three independent experiments.

Supplementary Figure 4. Screening eGFP expression after 48 h in growth arrested HepG2 cells as flexible linker length (GGGG(S))$_n$ is increased between DNA and NLS peptides is increased.

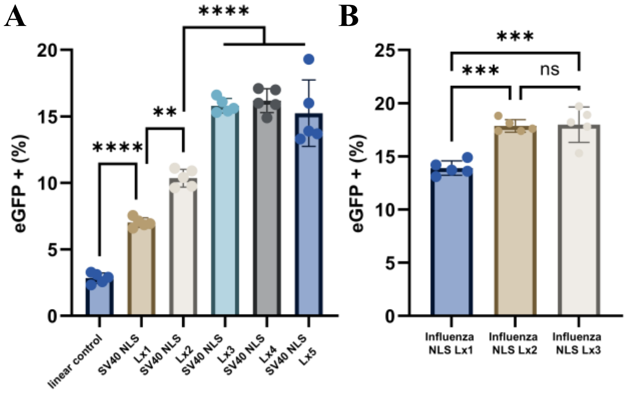

(A) shows data from increasing SV40 linker length, while (B) shows Influenza linker length. Data collected were quantified using flow cytometry and are presented as mean ± s.d. for $n = 5$ biologically independent experiments, individual data points are overlaid, 100 ng DNA transfected per condition. Statistical analysis was performed using one-way ANOVA with Tukey's multiple comparison (*$p \leq 0.05$, **$p \leq 0.01$, ***$p \leq 0.001$, ****$p \leq 0.0001$, ns $p > 0.05$). Source Data are provided as a Source Data file.

Supplementary Figure 5. Screening eGFP expression after 48 h in growth arrested HepG2 cells with dual end modified NLS peptides of varying linker length $(GGGG(S)_n$.

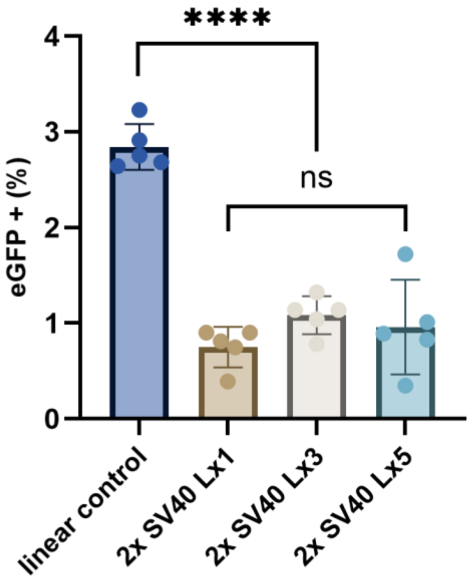

Data collected were quantified using flow cytometry and are presented as mean ± s.d. for $n = 5$ biologically independent experiments, individual data points are overlaid, 100 ng DNA transfected per condition. Statistical analysis was performed using one-way ANOVA with Tukey's multiple comparison ($*p \leq 0.05$, $**p \leq 0.01$, $***p \leq 0.001$, $****p \leq 0.0001$, ns $p > 0.05$). Source Data are provided as a Source Data file.

Supplementary Figure 6. Screening eGFP expression of electrostatically conjugated NLS peptides to DNA gene cassettes after 48 h in growth arrested HepG2 cells.

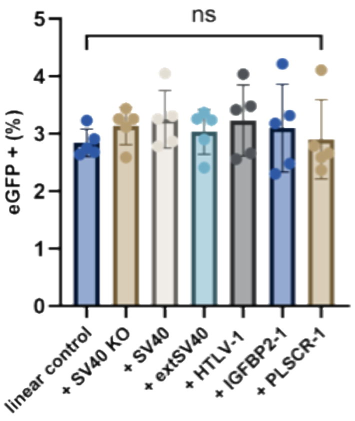

Data collected were quantified using flow cytometry and are presented as mean ± s.d. for $n \geq 5$ biologically independent experiments, individual data points are overlaid, 100 ng DNA transfected per condition. Statistical analysis was performed using one-way ANOVA with Tukey's multiple comparison (* $p \leq 0.05$, ** $p \leq 0.01$, *** $p \leq 0.001$, **** $p \leq 0.0001$, ns $p > 0.05$). Source Data are provided as a Source Data file.

Supplementary Figure 7. Total eGFP DNA cassette copy number delivered to growth-arrested HepG2 cells 48 h following lipofection.

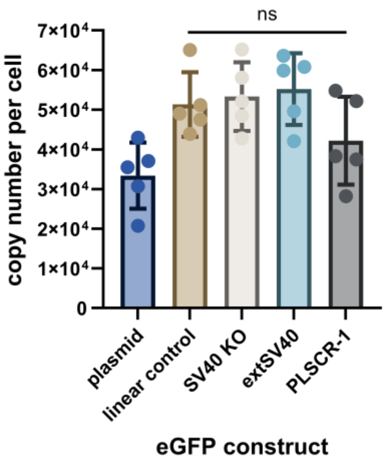

Data collected were quantified using qPCR, normalized against RPP30 as a housekeeping gene, and are presented as mean ± s.d. for $n = 5$ biologically independent experiments, with individual data points shown, 100 ng DNA transfected per condition. Statistical analysis was performed using one-way ANOVA with Tukey's multiple comparison ($*p \leq 0.05$, $**p \leq 0.01$, $***p \leq 0.001$, $****p \leq 0.0001$, ns $p > 0.05$). Source Data are provided as a Source Data file.

Supplementary Figure 8. Extranuclear, nuclear, and total eGFP DNA gene cassette copy numbers delivered to growth-arrested HepG2 cells 48 h following lipofection. The nuclear fraction of lysate was separated from the extranuclear fraction via centrifugation (see methods).

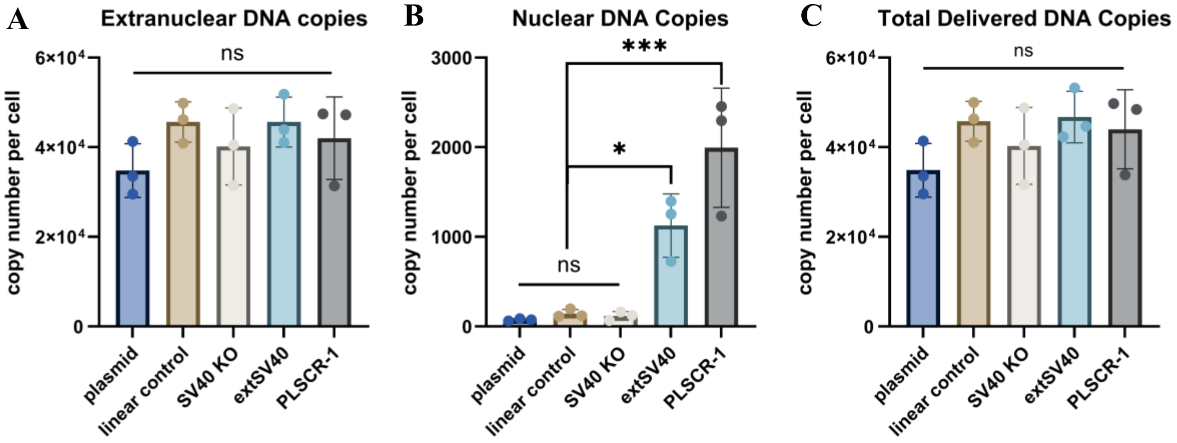

Data collected were quantified using qPCR, normalized against RPP30 as a housekeeping gene, and are presented as mean ± s.d. for $n = 3$ biologically independent experiments, with individual data points shown, 100 ng DNA transfected per condition. Nuclear DNA copies (B) are also presented in Figure 3d. Total delivered DNA copies (C) is a summation of extranuclear (A) and nuclear DNA copies (B). Statistical analysis was performed using one-way ANOVA with Tukey's multiple comparison (*$p \leq 0.05$, **$p \leq 0.01$, ***$p \leq 0.001$, ****$p \leq 0.0001$, ns $p > 0.05$). Source Data are provided as a Source Data file.

Supplementary Figure 9. mRNA transcripts copy numbers from eGFP DNA gene cassettes delivered to growth-arrested HepG2s via lipofection.

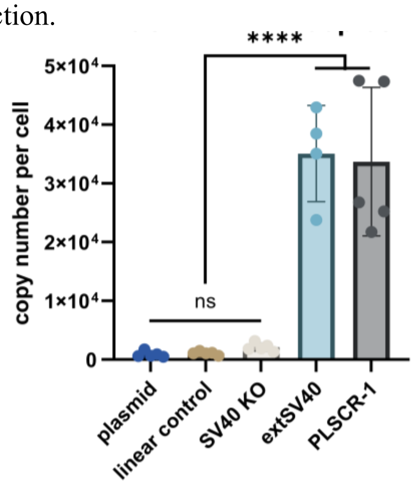

Data collected were quantified using qPCR, normalized against GAPDH as a housekeeping gene, and are presented as mean ± s.d. for $n \geq 4$ biologically independent experiments, with individual data points shown, 100 ng DNA transfected per condition. Statistical analysis was performed using one-way ANOVA with Tukey's multiple comparison (*$p \leq 0.05$, **$p \leq 0.01$, ***$p \leq 0.001$, ****$p \leq 0.0001$, ns $p > 0.05$). Source Data are provided as a Source Data file.

Supplementary Figure 10. Screening eGFP expression after 48 h in nocodazole growth arrested or actively dividing HepG2 cells with NLS-modified gene cassettes.

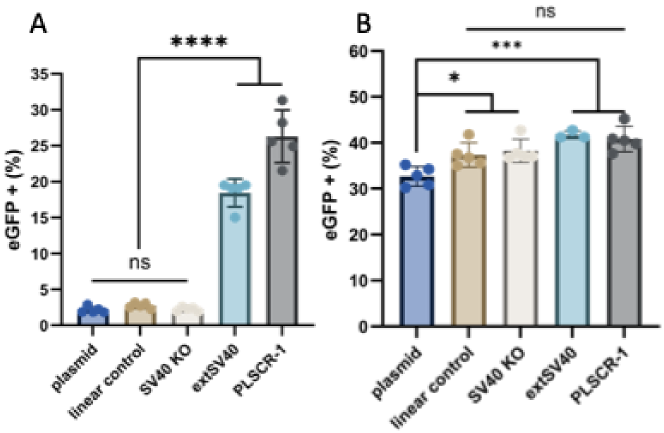

(A) Percent of nocodazole growth-arrested HepG2s with eGFP fluorescence 48 hours after transfection. (B) Percent of actively dividing HepG2s with eGFP fluorescence 48 hours after transfection. Data collected were quantified using flow cytometry and are presented as mean ± s.d. for $n \geq 3$ biologically independent experiments, individual data points are overlaid, 100 ng DNA transfected per condition. Statistical analysis was performed using one-way ANOVA with Tukey's multiple comparison (*$p \leq 0.05$, **$p \leq 0.01$, ***$p \leq 0.001$, ****$p \leq 0.0001$, ns $p > 0.05$). Source Data are provided as a Source Data file.

Supplementary Figure 11. Example flow cytometry gating scheme used for eGFP expression in HepG2 cells.

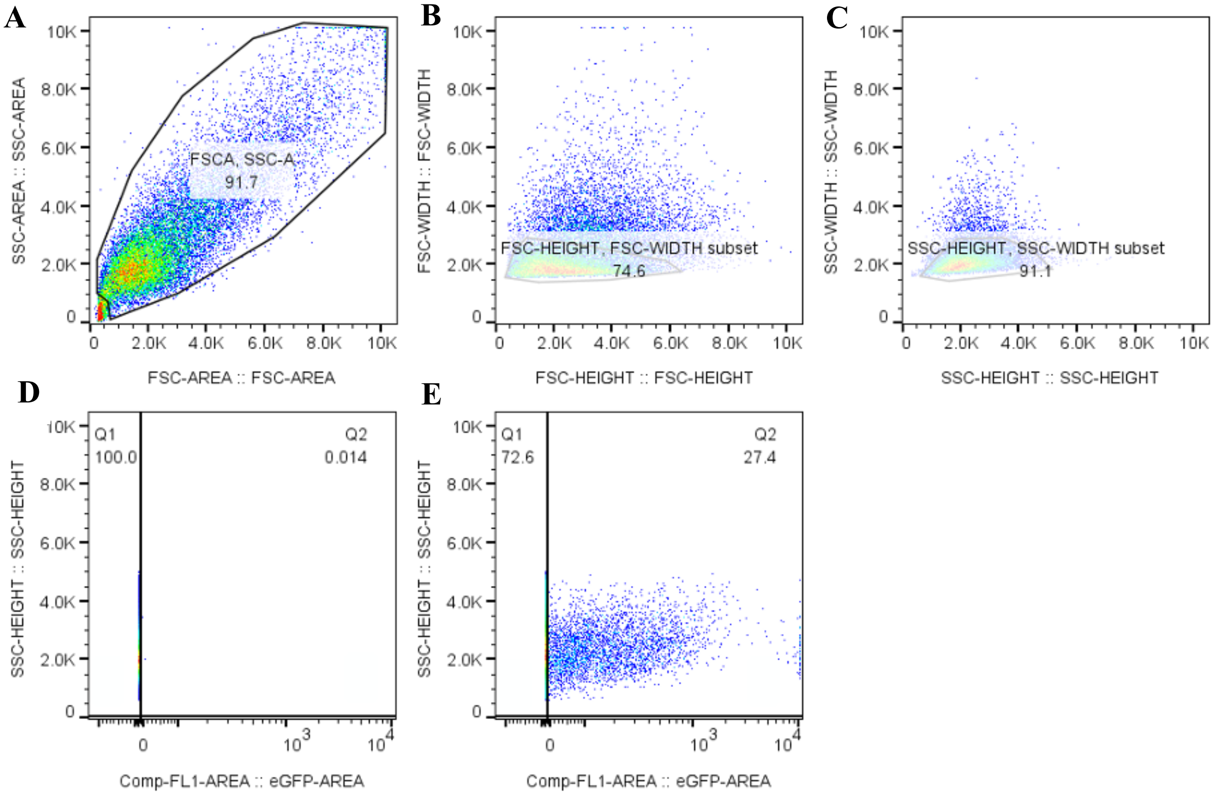

(A-C) Cell debris was gated out using side scattering area against forward scattering area, and doublets were gated out using width against forward and side scattering height. Cells were then assessed for eGFP expression. In this example, negative (untransfected HepG2 cells) control (D) and PLSCR-1 NLS modified eGFP gene cassette (E) were investigated for eGFP expression (x-axis), where cells can be eGFP negative (Q1) or eGFP positive (Q2). The negative control was used to set the thresholding gate for eGFP positive cells as untransfected cells would have minimal fluorescence following elimination of cell debris which may be autoflourescent.

Supplementary Figure 12. Example flow cytometry gating scheme used for eGFP expression in HEK293T cells.

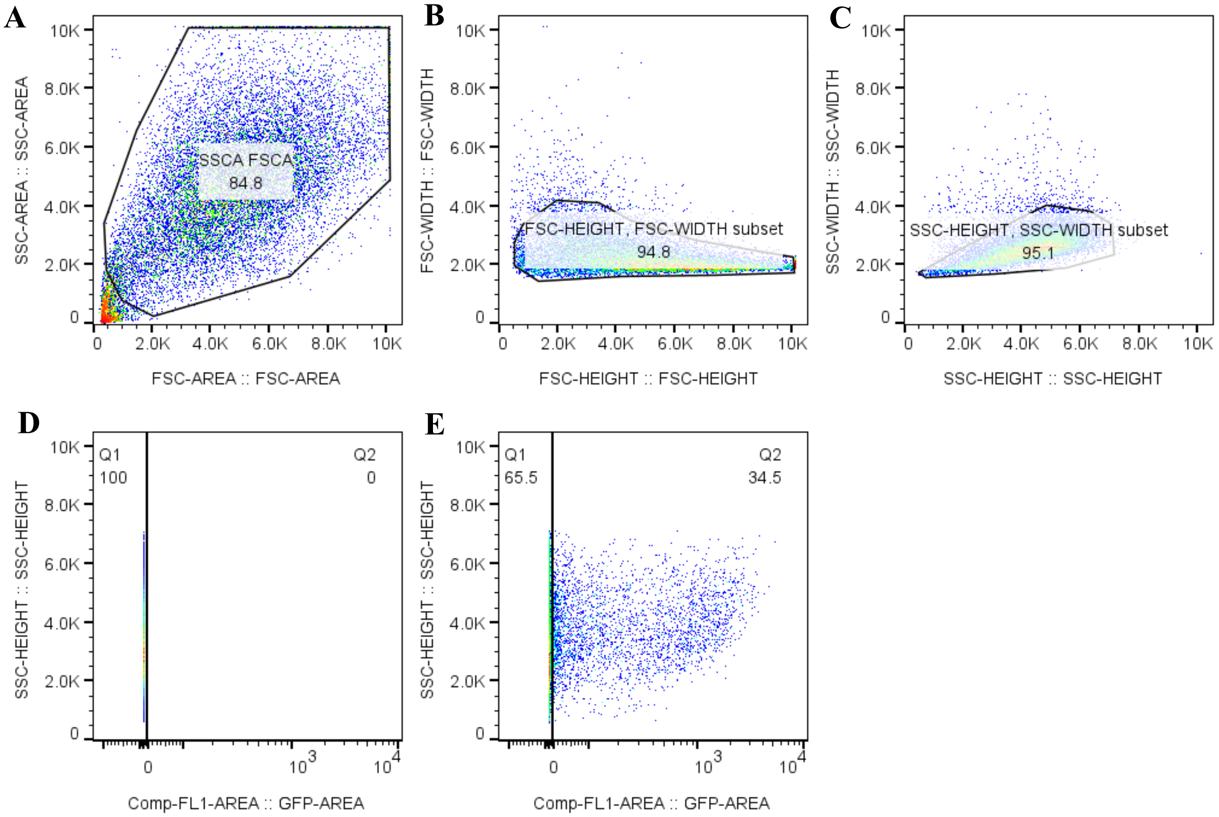

(A-C) Cell debris was gated out using side scattering area against forward scattering area, and doublets were gated out using width against forward and side scattering height. Cells were then assessed for eGFP expression. In this example, negative (untransfected HEK293T cells) control (D) and PLSCR-1 NLS modified eGFP gene cassette (E) were investigated for eGFP expression (x-axis), where cells can be eGFP negative (Q1) or eGFP positive (Q2). The negative control was used to set the thresholding gate for eGFP positive cells as untransfected cells would have minimal fluorescence following elimination of cell debris which may be autoflourescent.

Supplementary Figure 13. Example flow cytometry gating scheme used for eGFP expression in AC16 cells.

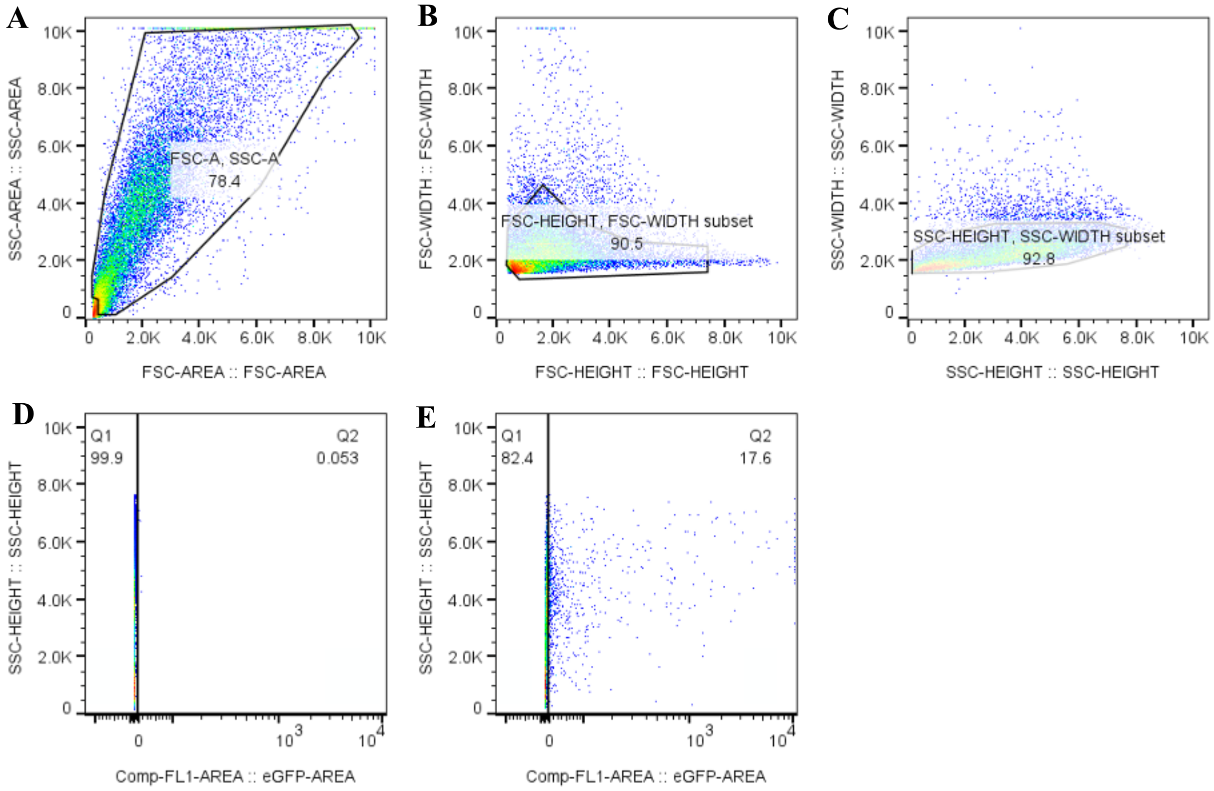

(A-C) Cell debris was gated out using side scattering area against forward scattering area, and doublets were gated out using width against forward and side scattering height. Cells were then assessed for eGFP expression. In this example, negative (untransfected AC16 cells) control (D) and PLSCR-1 NLS modified eGFP gene cassette (E) were investigated for eGFP expression (x-axis), where cells can be eGFP negative (Q1) or eGFP positive (Q2). The negative control was used to set the thresholding gate for eGFP positive cells as untransfected cells would have minimal fluorescence following elimination of cell debris which may be autoflourescent.

Supplementary Figure 14. Representative epifluorescence microscopy images showing 24 h NLS-eGFP expression in growth arrested HepG2 cells under various concentrations of importazole.

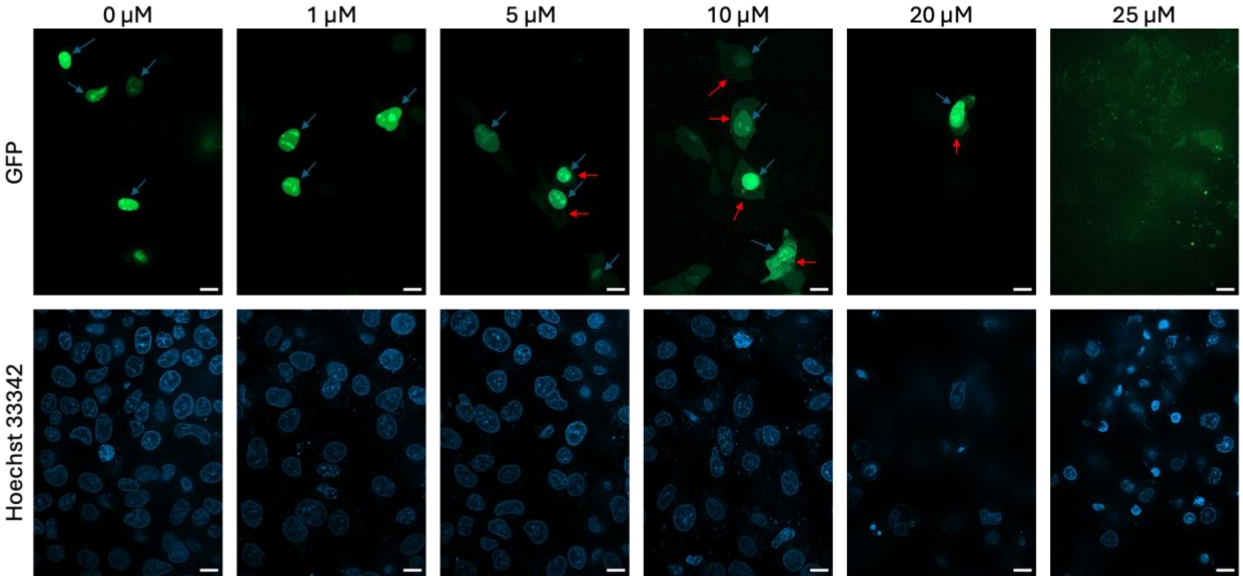

Blue arrows indicate NLS-eGFP localized to the nucleus, while red arrows highlight NLS-eGFP leakage into the cytosol due to importin-β inhibition by importazole. Among the tested concentrations, 10 μM importazole exhibited the strongest importin-β inhibition while maintaining cell viability by intact nuclei. Higher concentrations (20 and 25 μM) resulted in increased cytotoxicity evidenced by the lack of intact nuclei (Hoechst 33342 staining). Expression was assessed only at 24 h and not longer due to the cytotoxic properties of importazole. Scale bar: 10 μm. Shown are representative images from 3 independent experiments.

Supplementary Figure 15. High resolution mass spectrum of preQ$_1$-DBCO.

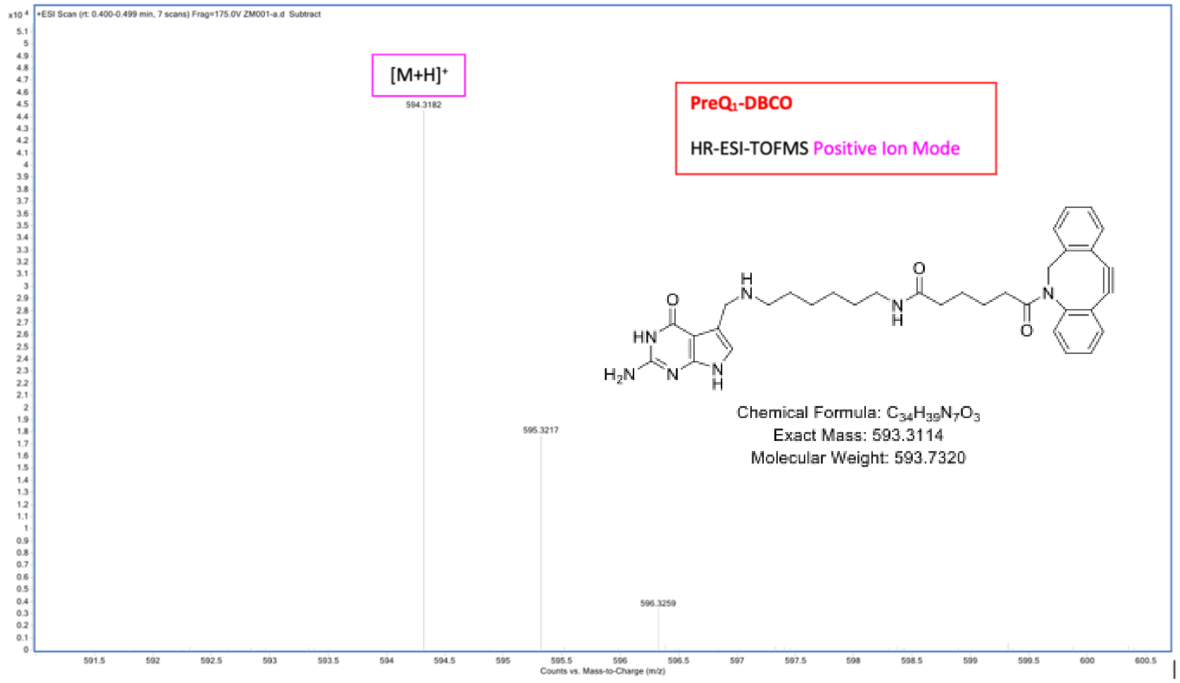

Mass Measured	Theo. Mass	Delta (ppm)	Composition
594.3182	594.3187	-0.8	$[C_{34}H_{40}N_7O_3]^+$

HRMS spectrum was acquired in +ESI mode. Agilent MassHunter workstation was used to assign ions and verify mass. Representative HRMS spectrum from two independent experiments.

Supplementary Figure 16. High resolution mass spectrum of SV40 Lx1 NLS peptide.

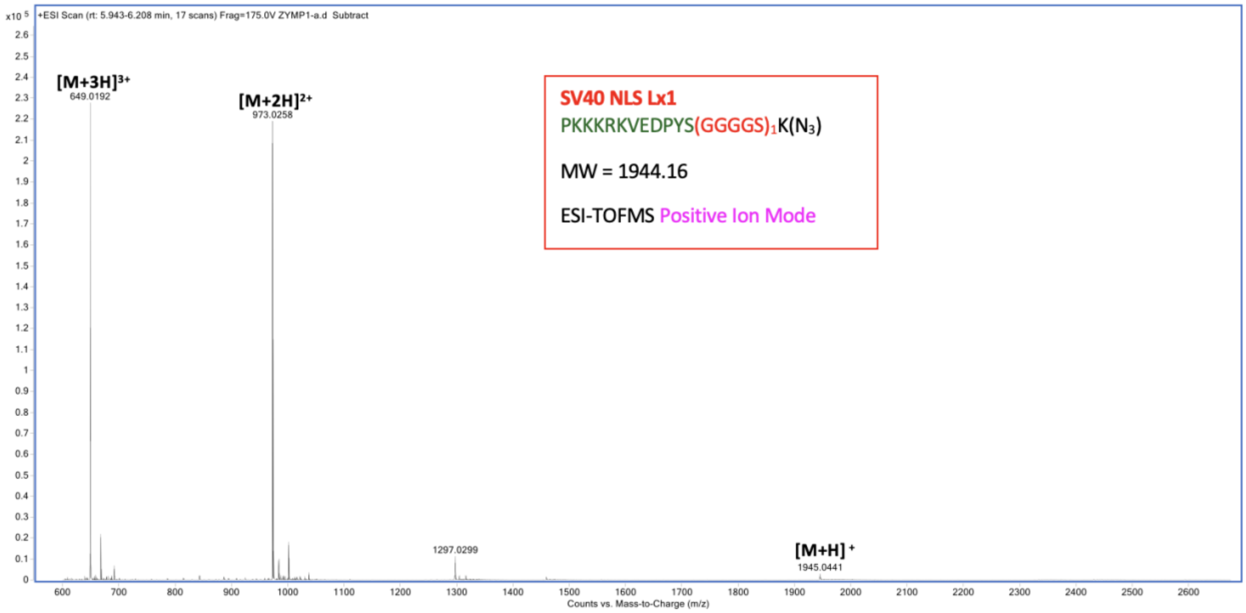

HRMS spectrum was acquired in +ESI mode. Agilent MassHunter workstation was used to assign ions and verify mass. Peptide sequence and molecular weight is provided. Representative HRMS spectrum from three independent experiments.

Supplementary Figure 17. High resolution mass spectrum of SV40 Lx2 NLS peptide.

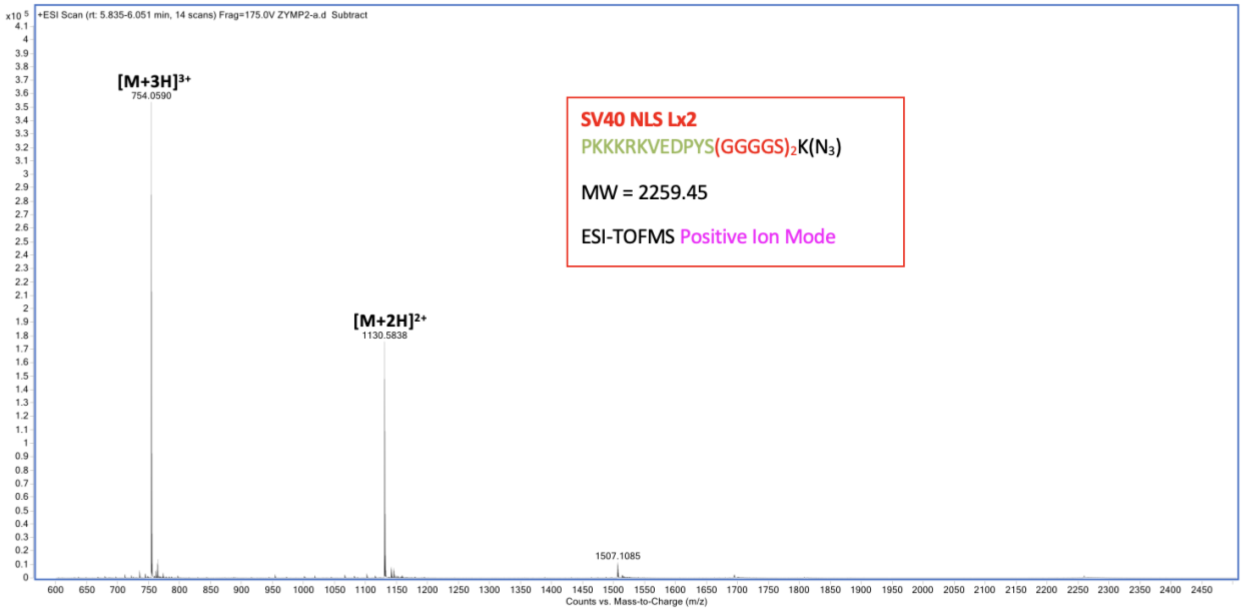

HRMS spectrum was acquired in +ESI mode. Agilent MassHunter workstation was used to assign ions and verify mass. Peptide sequence and molecular weight is provided. Representative HRMS spectrum from three independent experiments.

Supplementary Figure 18. High resolution mass spectrum of SV40 Lx3 NLS peptide.

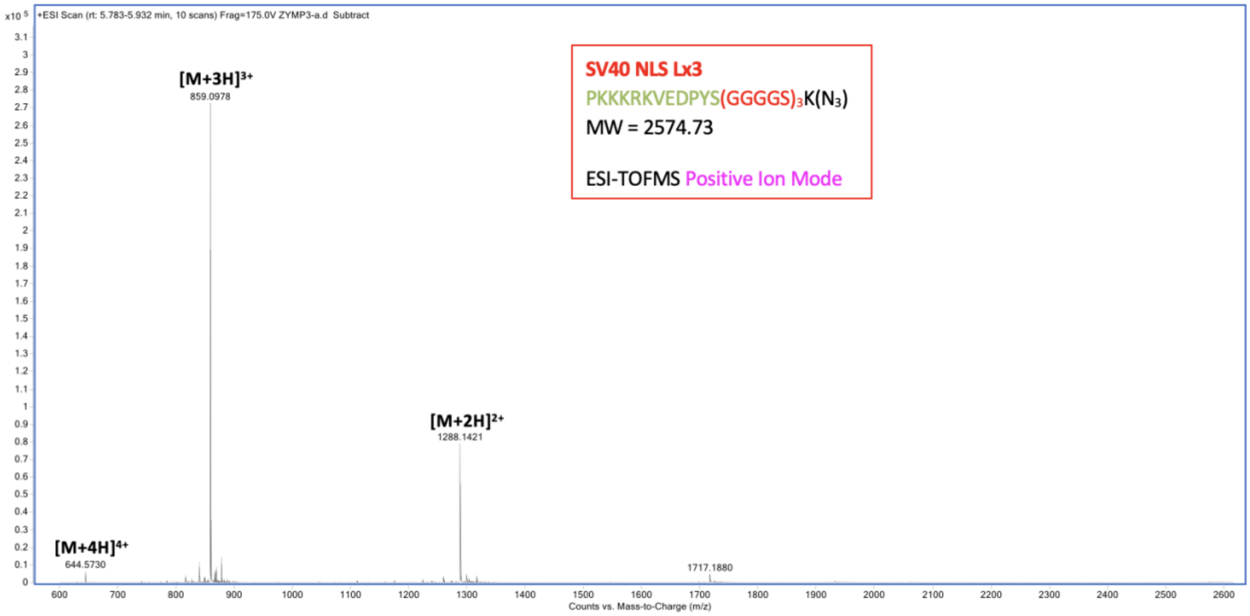

HRMS spectrum was acquired in +ESI mode. Agilent MassHunter workstation was used to assign ions and verify mass. Peptide sequence and molecular weight is provided. Representative HRMS spectrum from three independent experiments.

Supplementary Figure 19. High resolution mass spectrum of SV40 Lx4 NLS peptide.

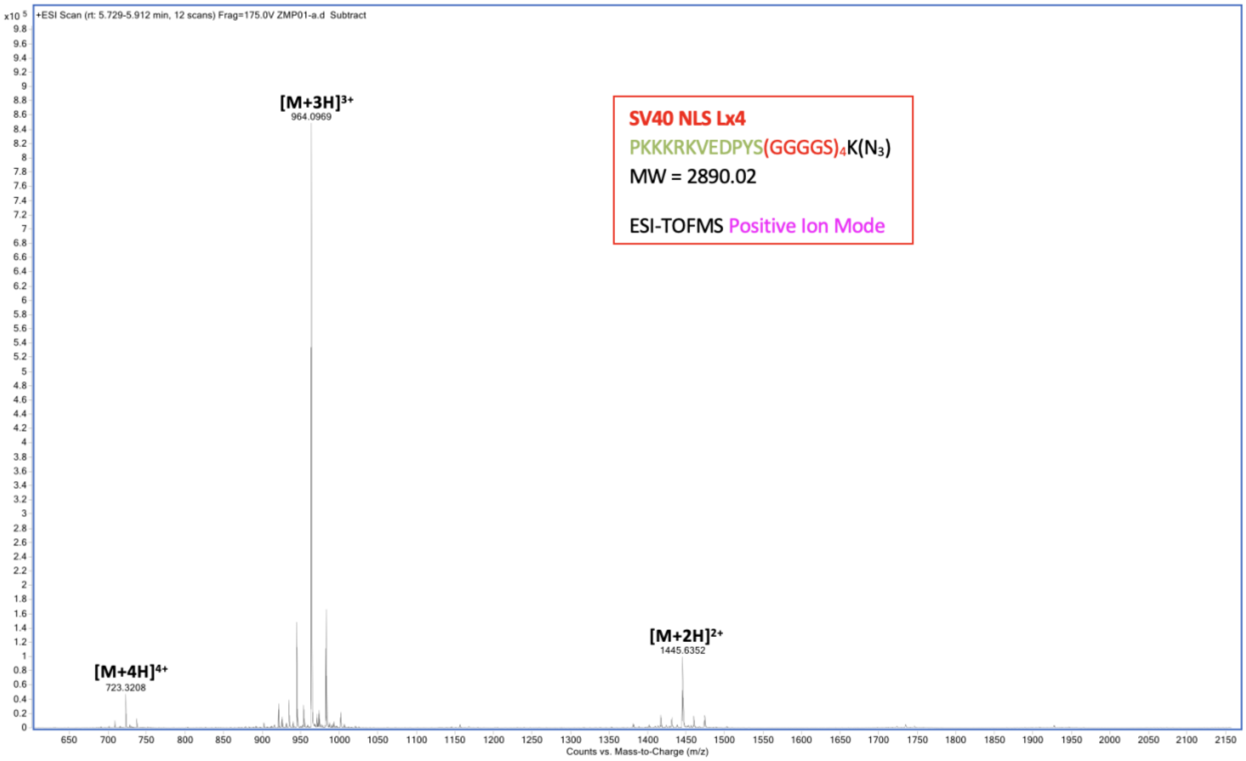

HRMS spectrum was acquired in +ESI mode. Agilent MassHunter workstation was used to assign ions and verify mass. Peptide sequence and molecular weight is provided. Representative HRMS spectrum from three independent experiments.

Supplementary Figure 20. High resolution mass spectrum of SV40 Lx5 NLS peptide.

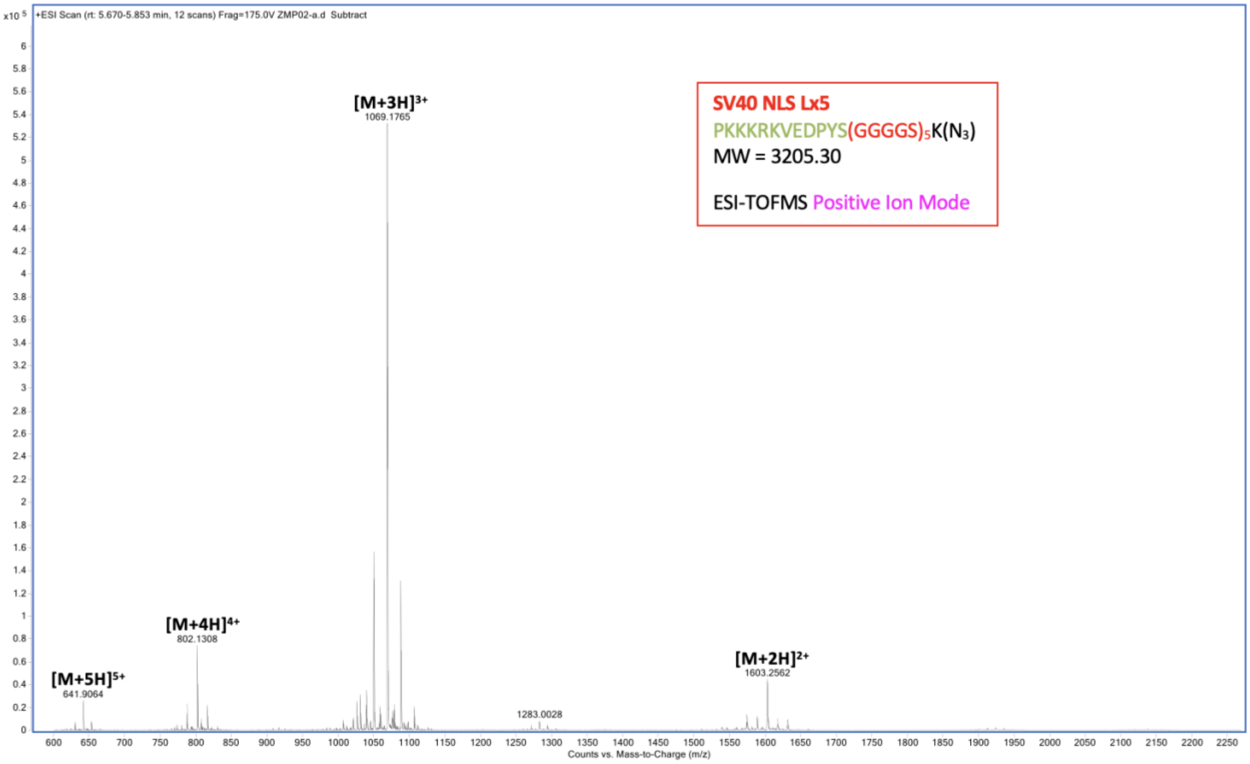

HRMS spectrum was acquired in +ESI mode. Agilent MassHunter workstation was used to assign ions and verify mass. Peptide sequence and molecular weight is provided. Representative HRMS spectrum from three independent experiments.

Supplementary Figure 21. High resolution mass spectrum of Influenza A Virus A NP Lx2 NLS peptide.

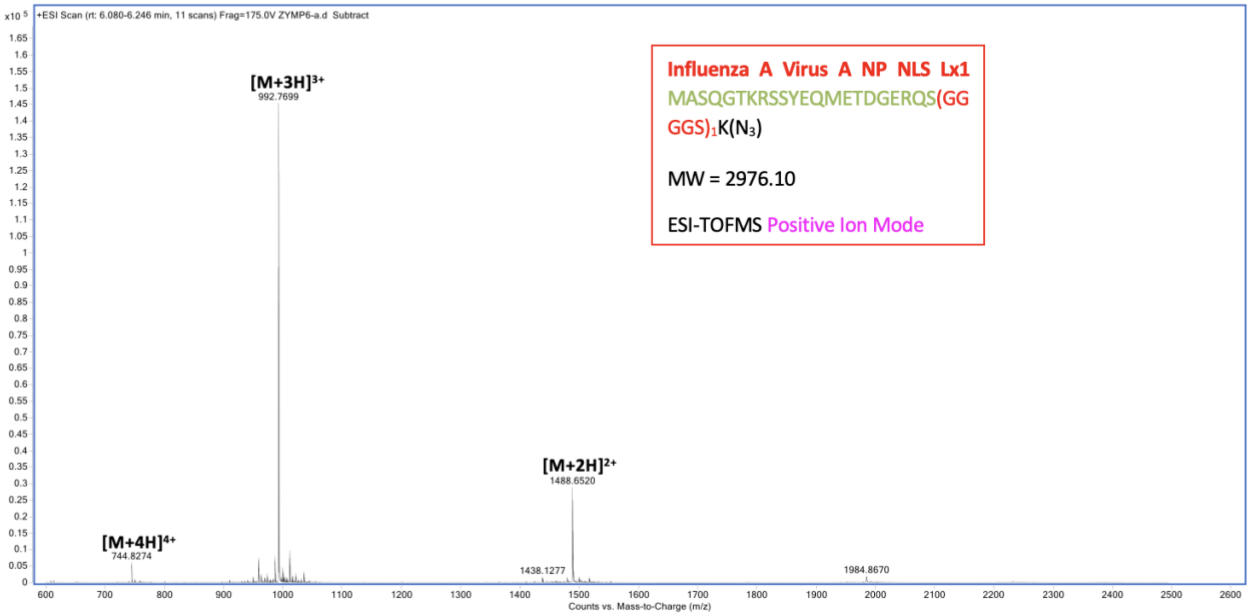

HRMS spectrum was acquired in +ESI mode. Agilent MassHunter workstation was used to assign ions and verify mass. Peptide sequence and molecular weight is provided. Representative HRMS spectrum from three independent experiments.

Supplementary Figure 22. High resolution mass spectrum of Influenza A Virus A NP Lx2 peptide.

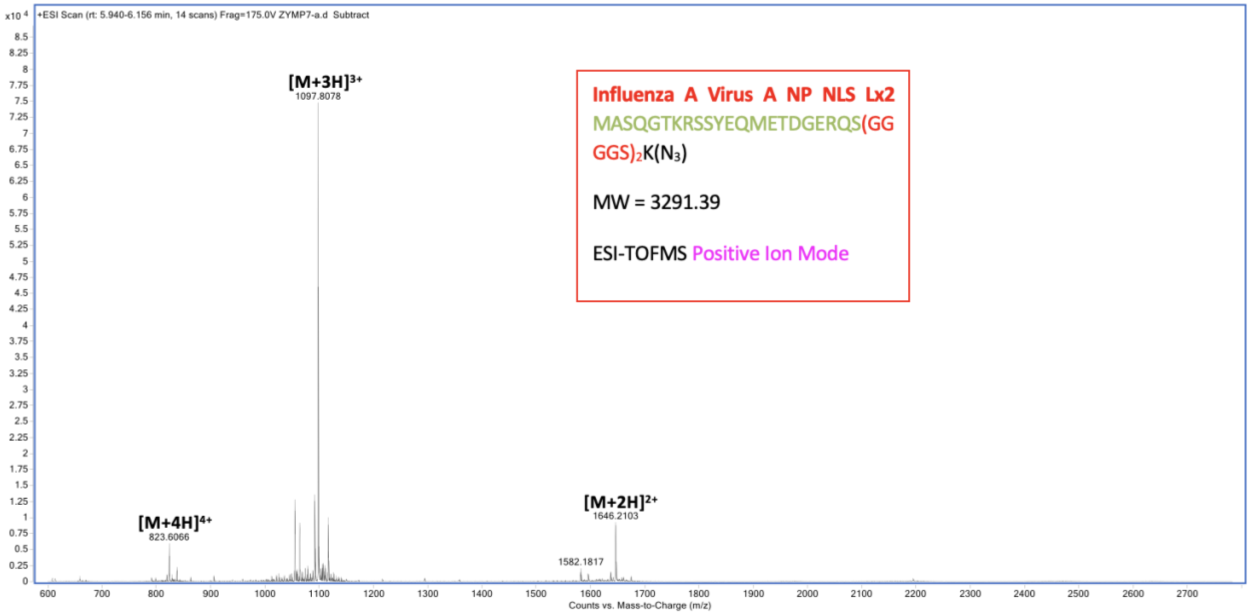

HRMS spectrum was acquired in +ESI mode. Agilent MassHunter workstation was used to assign ions and verify mass. Peptide sequence and molecular weight is provided. Representative HRMS spectrum from three independent experiments.

Supplementary Figure 23. High resolution mass spectrum of Influenza A Virus A NP Lx3 peptide.

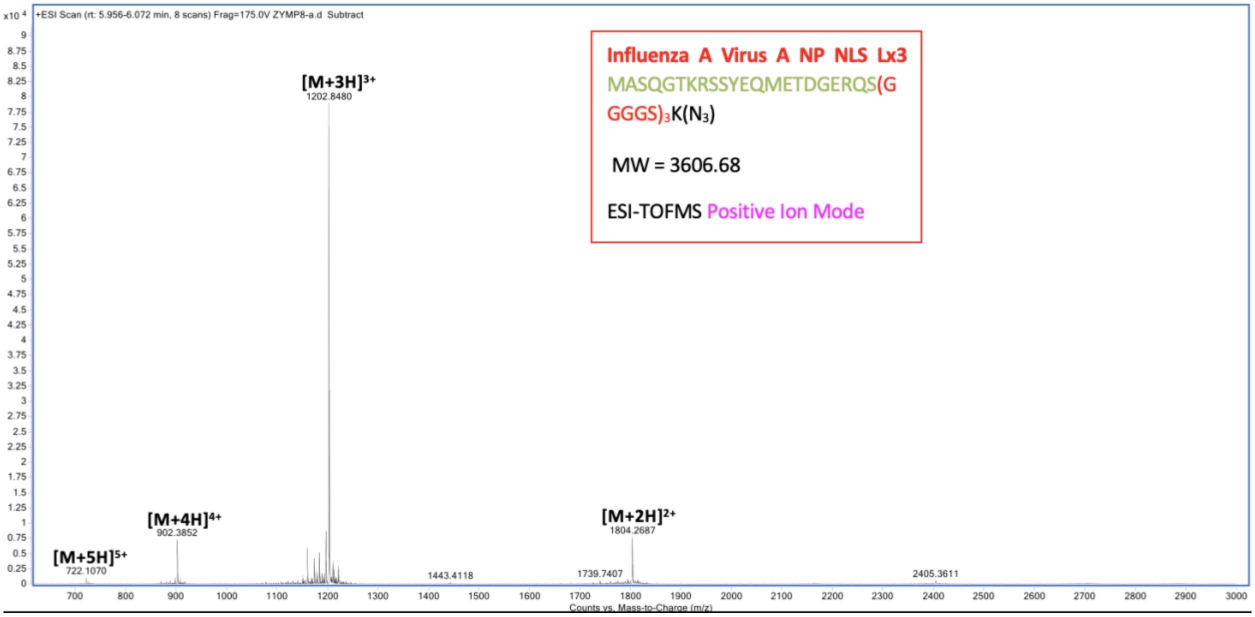

HRMS spectrum was acquired in +ESI mode. Agilent MassHunter workstation was used to assign ions and verify mass. Peptide sequence and molecular weight is provided. Representative HRMS spectrum from three independent experiments.

Supplementary Figure 24. High resolution mass spectrum of SV40 KO Lx3 peptide.

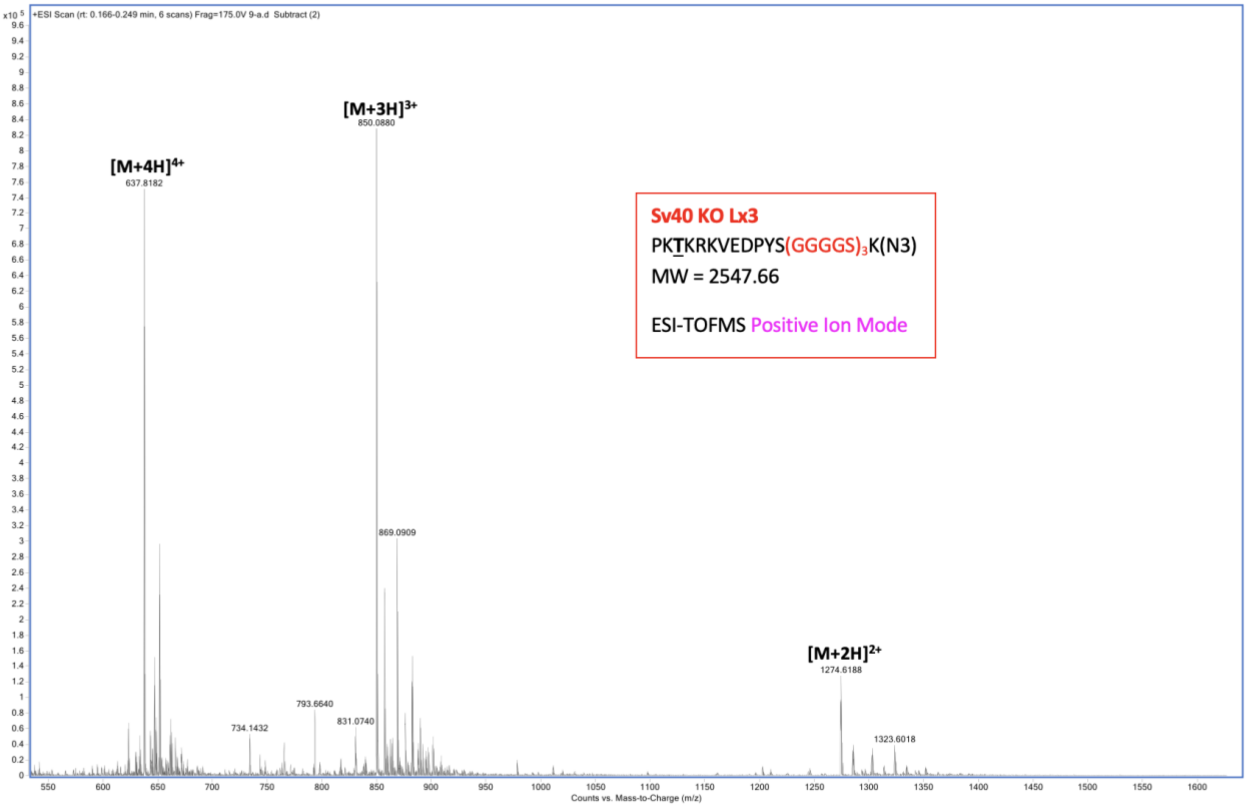

HRMS spectrum was acquired in +ESI mode. Agilent MassHunter workstation was used to assign ions and verify mass. Peptide sequence and molecular weight is provided. Representative HRMS spectrum from two independent experiments.

Supplementary Figure 25. High resolution mass spectrum of extSV40 Lx3 NLS peptide.

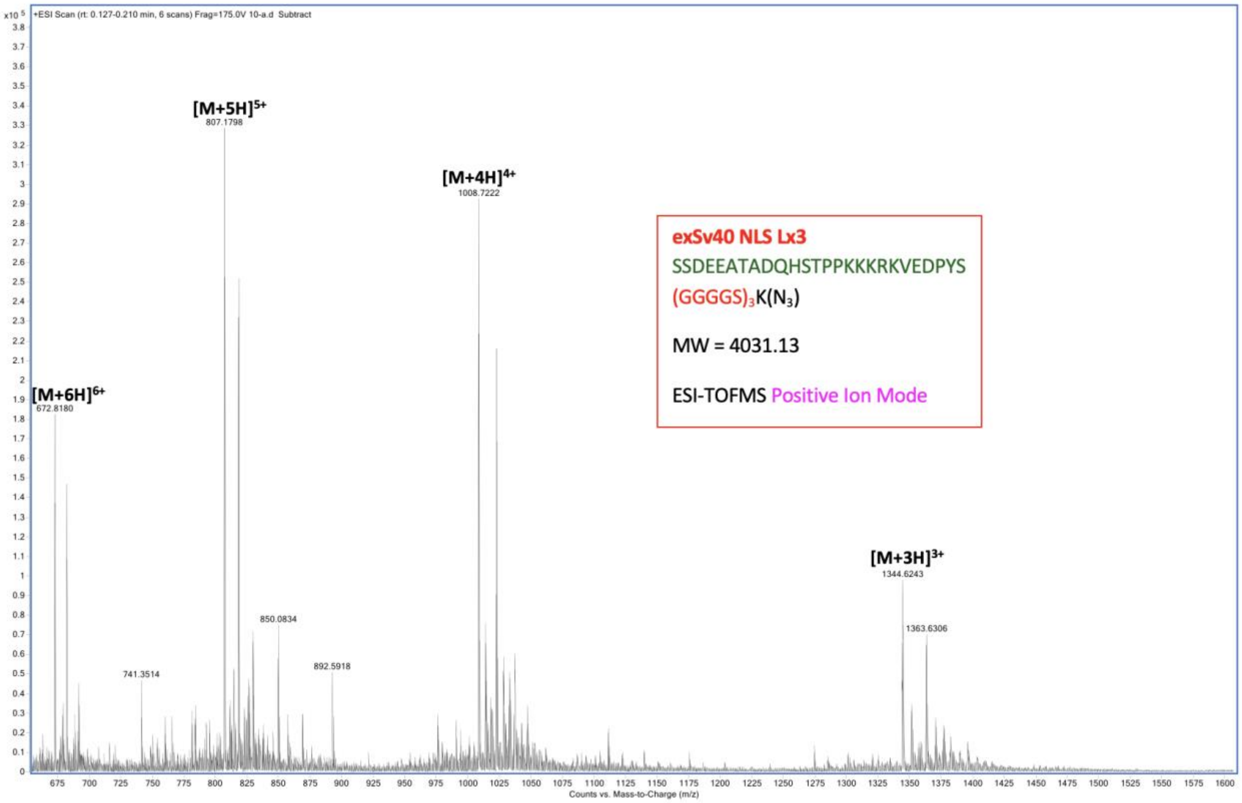

HRMS spectrum was acquired in +ESI mode. Agilent MassHunter workstation was used to assign ions and verify mass. Peptide sequence and molecular weight is provided. Representative HRMS spectrum from two independent experiments.

Supplementary Figure 26. High resolution mass spectrum of BVP Lx3 NLS peptide.

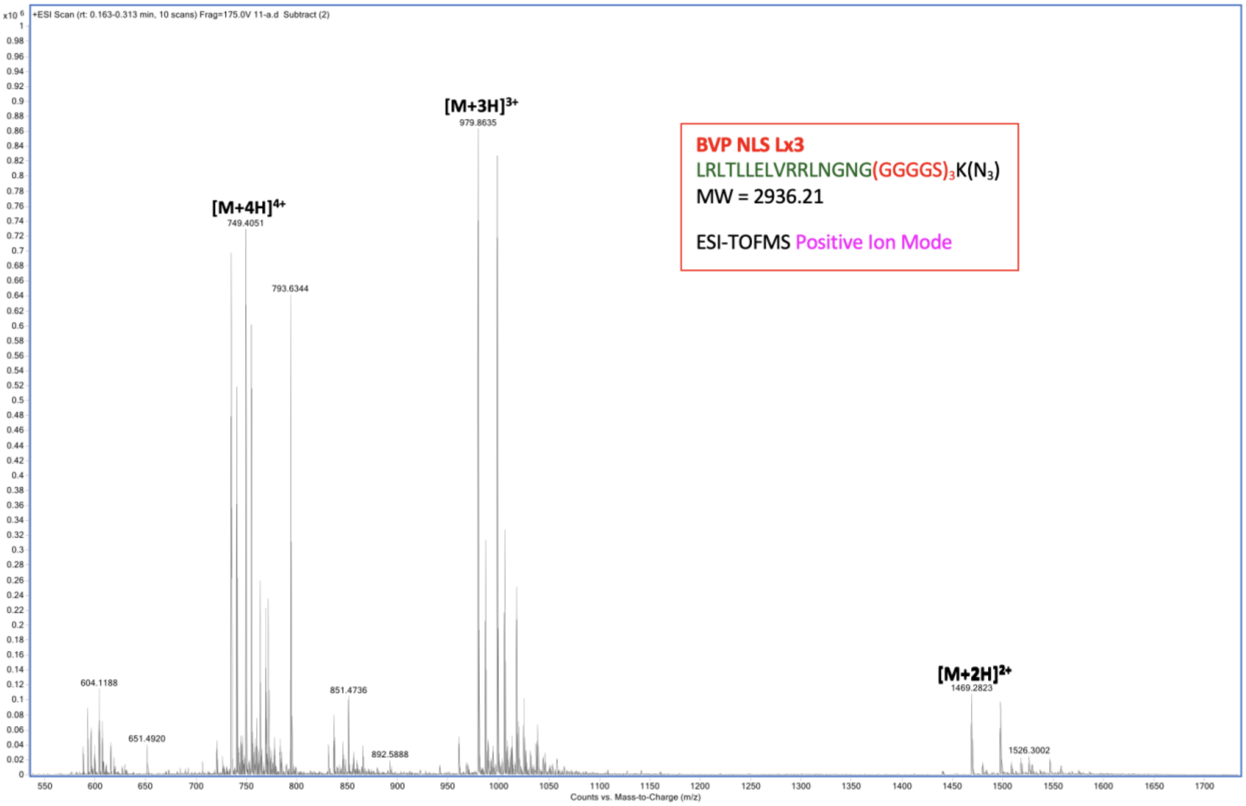

HRMS spectrum was acquired in +ESI mode. Agilent MassHunter workstation was used to assign ions and verify mass. Peptide sequence and molecular weight is provided. Representative HRMS spectrum from two independent experiments.

Supplementary Figure 27. High resolution mass spectrum of HTLV-1 Lx3 NLS peptide.

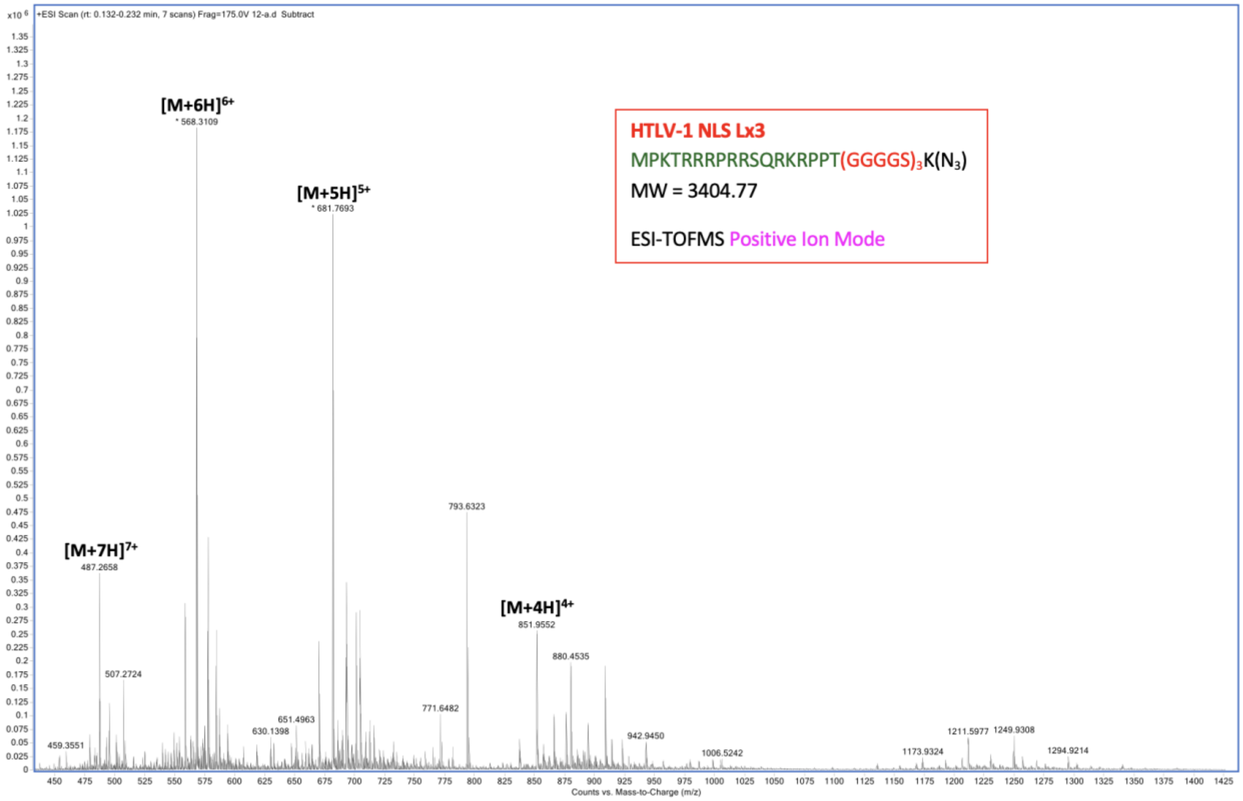

HRMS spectrum was acquired in +ESI mode. Agilent MassHunter workstation was used to assign ions and verify mass. Peptide sequence and molecular weight is provided. Representative HRMS spectrum from two independent experiments.

Supplementary Figure 28. High resolution mass spectrum of HTLV-1 Lx3 NLS peptide.

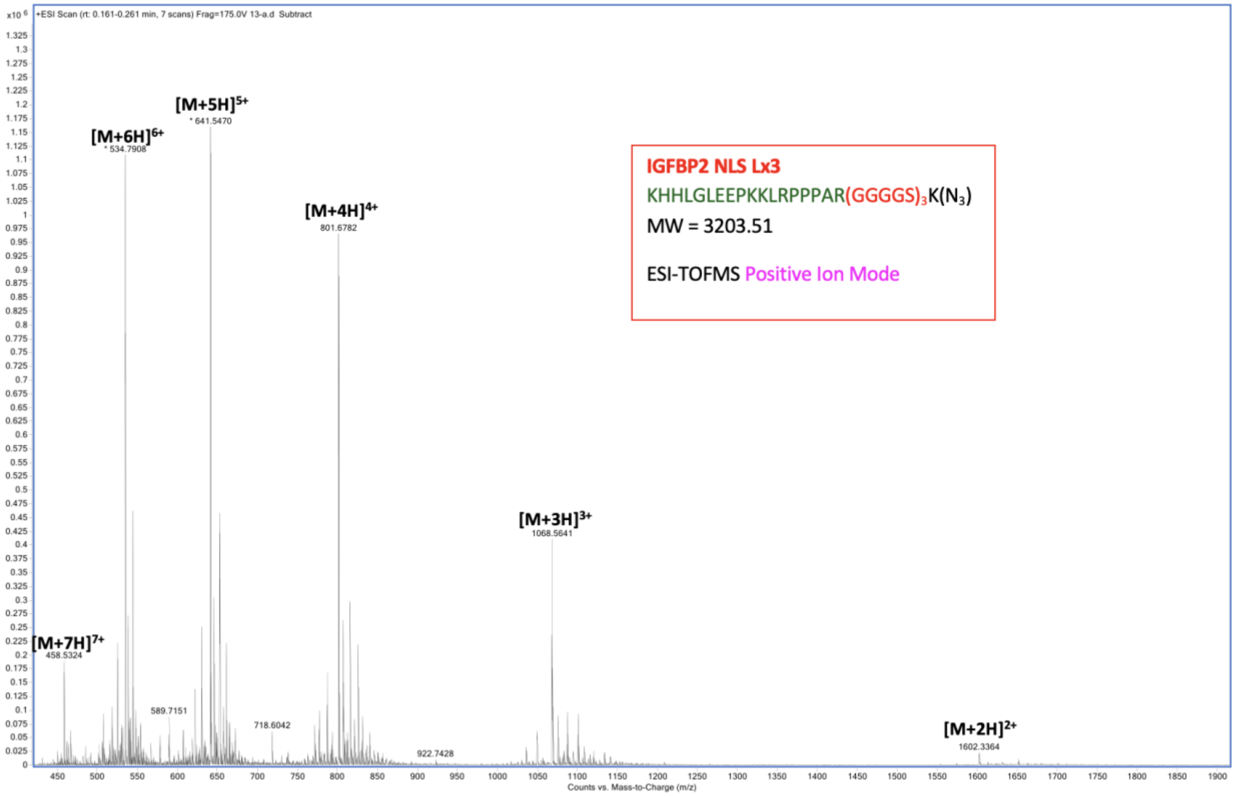

HRMS spectrum was acquired in +ESI mode. Agilent MassHunter workstation was used to assign ions and verify mass. Peptide sequence and molecular weight is provided. Representative HRMS spectrum from two independent experiments.

Supplementary Figure 29. High resolution mass spectrum of Hrp-1 Lx3 NLS peptide.

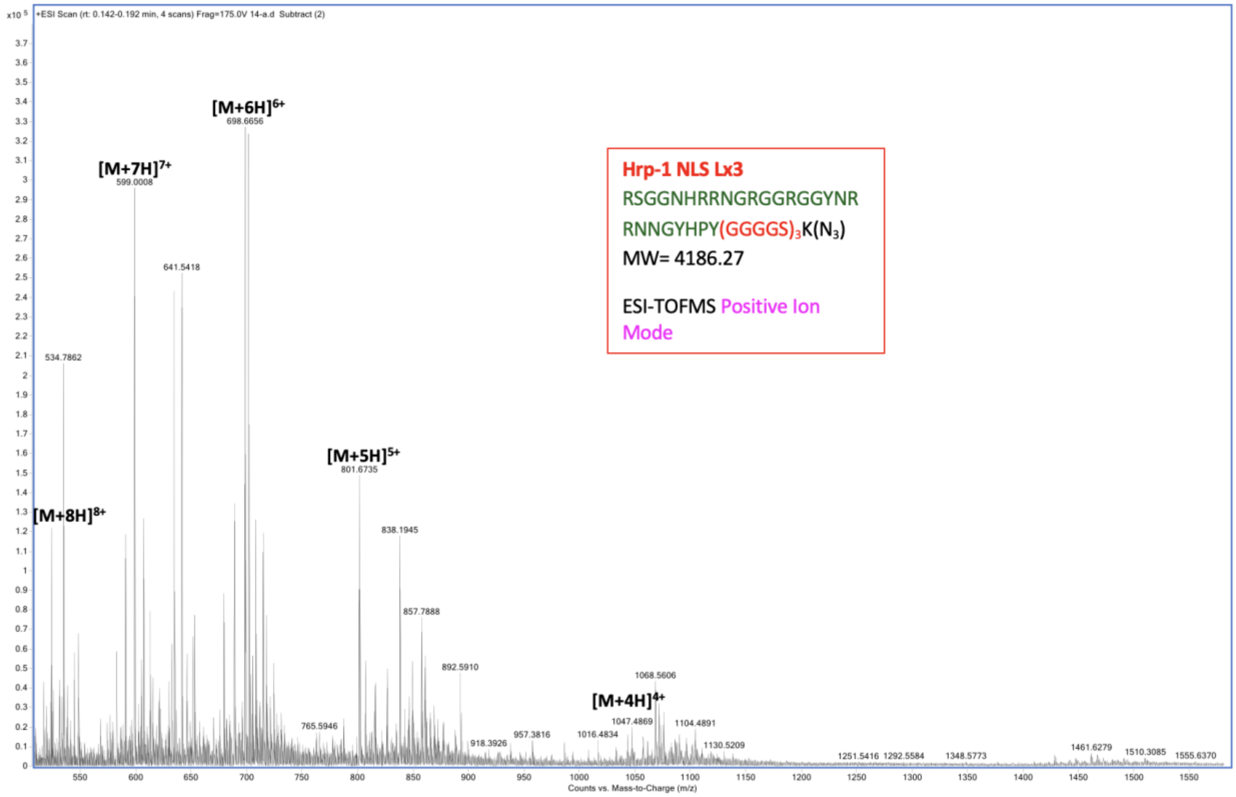

HRMS spectrum was acquired in +ESI mode. Agilent MassHunter workstation was used to assign ions and verify mass. Peptide sequence and molecular weight is provided. Representative HRMS spectrum from two independent experiments.

Supplementary Figure 30. High resolution mass spectrum of PLSCR-1 Lx3 NLS peptide.

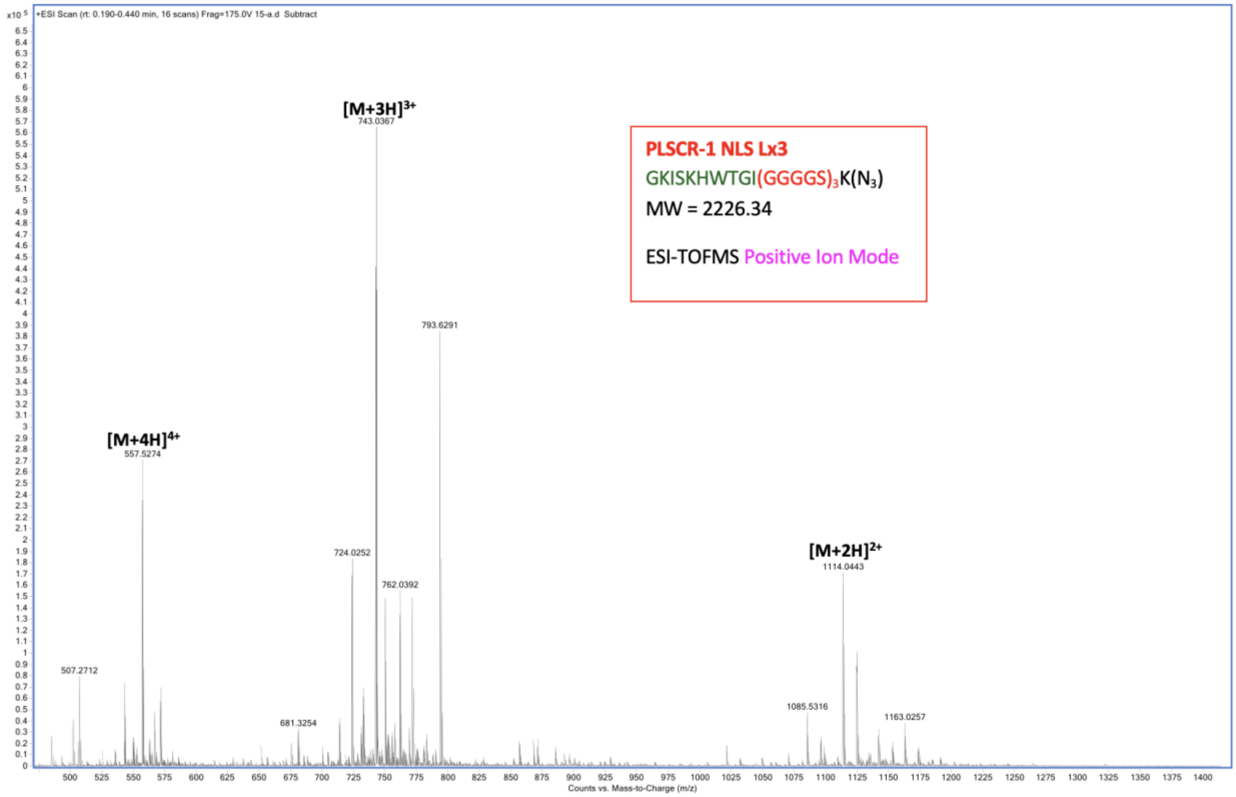

HRMS spectrum was acquired in +ESI mode. Agilent MassHunter workstation was used to assign ions and verify mass. Peptide sequence and molecular weight is provided. Representative HRMS spectrum from two independent experiments.

Supplementary Figure 31. High resolution mass spectrum of MluI 170 PP oligonucleotide-SV40 Lx3 NLS conjugate.

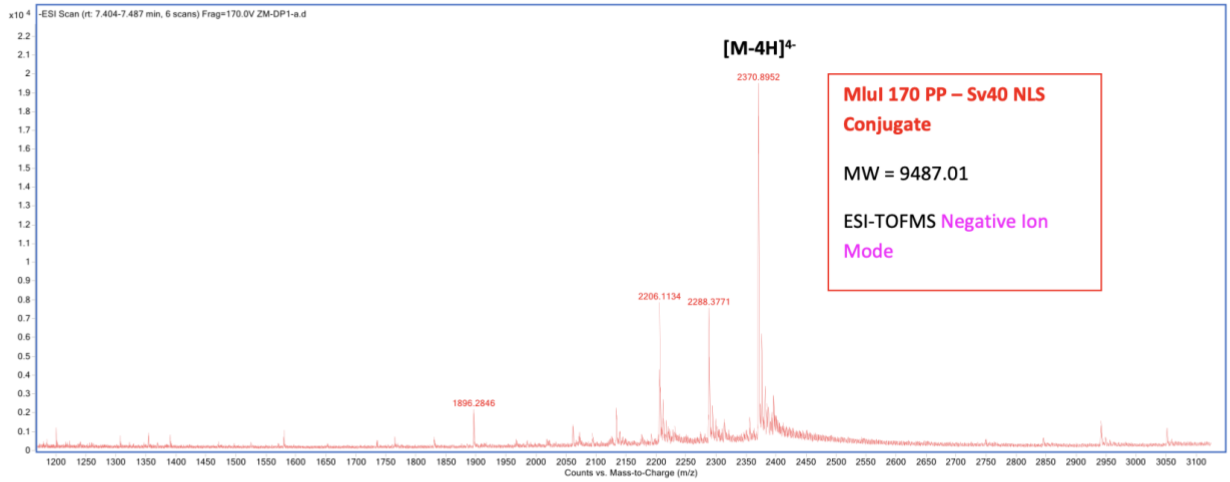

HRMS spectrum was acquired in -ESI mode. Agilent MassHunter workstation was used to assign ions and verify mass. Peptide sequence and molecular weight is provided. Representative HRMS spectrum from three independent experiments.

Supplementary Figure 32. High resolution mass spectrum of MluI 170 PP oligonucleotide-SV40 KO Lx3 conjugate.

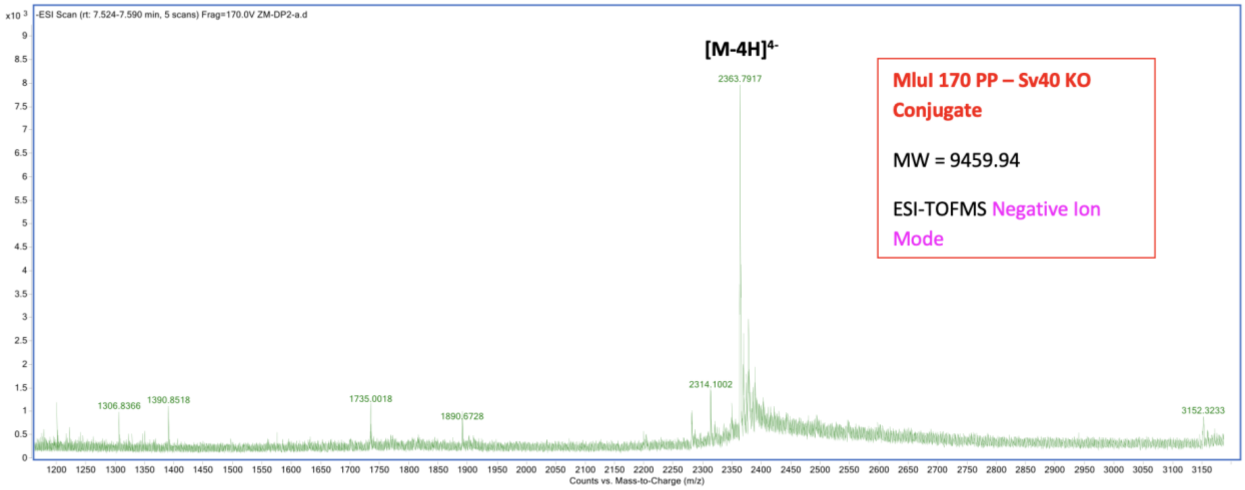

HRMS spectrum was acquired in -ESI mode. Agilent MassHunter workstation was used to assign ions and verify mass. Peptide sequence and molecular weight is provided. Representative HRMS spectrum from three independent experiments.

Supplementary Figure 33. High resolution mass spectrum of MluI 170 PP oligonucleotide-PLSCR Lx3 NLS conjugate.

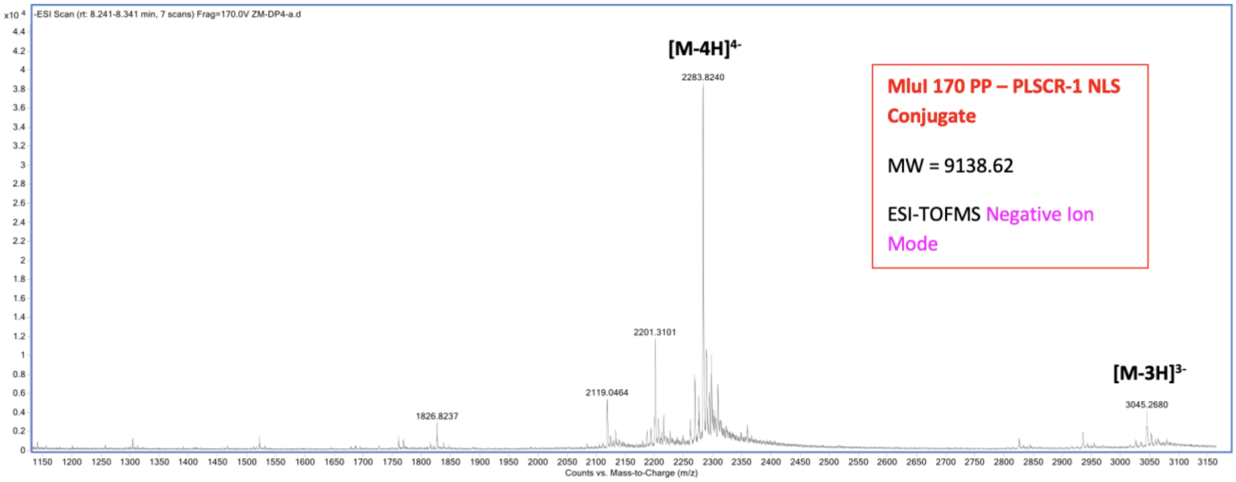

HRMS spectrum was acquired in -ESI mode. Agilent MassHunter workstation was used to assign ions and verify mass. Peptide sequence and molecular weight is provided. Representative HRMS spectrum from three independent experiments.

Supplementary Figure 34. Overview of $preQ_1$-DBCO synthesis from commercially purchased precursors.

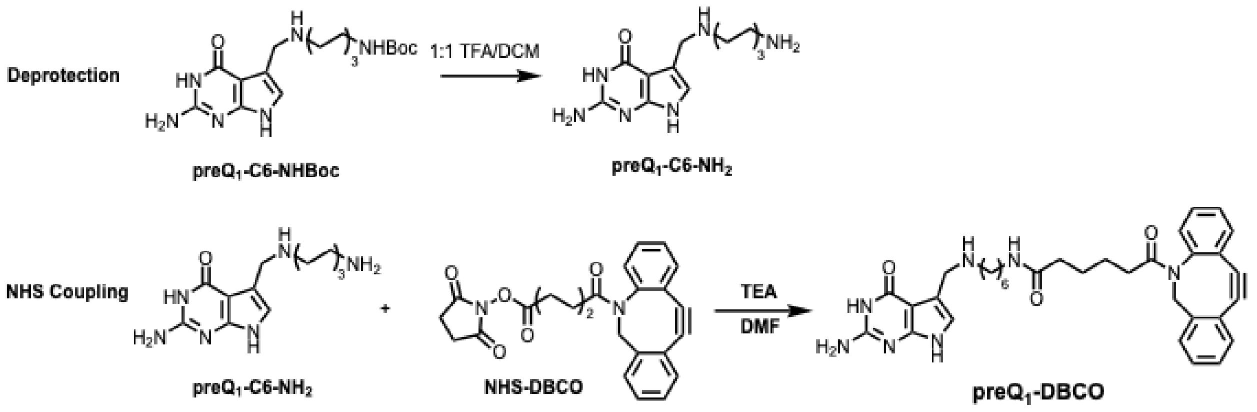

$PreQ_1$-DBCO was synthesized as previously described.[1,4] Briefly, a tert-butyloxycarbonyl (boc) protecting group from a $preQ_1$ derivative was removed and the free amine was conjugated to NHS-DBCO from BroadPharm. The boc protected starting material was deprotected in 1:1 TFA/DCM for one hour. TFA was neutralized three times by washing with 10% TEA in DCM, roto-evaporating the solution between each wash. The resulting crude oil was dissolved in dry DMF and TEA was added dropwise. NHS-DBCO was dissolved in dry DMF and added to $preQ_1$-C6-NH_2 in DMF dropwise with stirring. The reaction proceeded for two hours at room temperature. Then, DMF was evaporated, and the crude product was purified by HPLC. The final product was confirmed by HRMS (expected mass = 593.3114, measured mass = 594.3184 for H^+ ion).

Supplementary Figure 35. Overview of generation of DNA oligonucleotide–peptide conjugates from DNA-DBCO and azido-peptides.

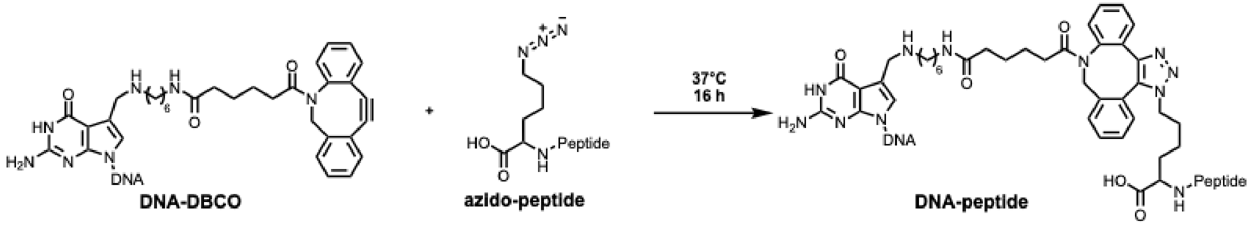

Generation of DNA oligonucleotide–peptide conjugates via strain-promoted azide-alkyne click chemistry. A 1:5 ratio of DNA-DBCO and azido-peptide were mixed for 16 h at 37°C to generate DNA-peptide conjugates. Conjugates were purified using the Oligo Clean & Concentrator kit (Zymo Research, D4061), eluting in pure water. Conjugates were confirmed by Urea-PAGE gel shifts and HRMS.

Supplementary References

1. Tota, E. M. & Devaraj, N. K. Site-Specific Covalent Labeling of DNA Substrates by an RNA Transglycosylase. *Journal of the American Chemical Society* **145**, 8099–8106 (2023).
2. Liang, G.-T. *et al.* Enhanced small green fluorescent proteins as a multisensing platform for biosensor development. *Front. Bioeng. Biotechnol.* **10**, (2022).
3. Cowan, Q. T. *et al.* Development of multiplexed orthogonal base editor (MOBE) systems. *Nat Biotechnol* 1–15 (2024) doi:10.1038/s41587-024-02240-0.
4. Alexander, S. C., Busby, K. N., Cole, C. M., Zhou, C. Y. & Devaraj, N. K. Site-specific covalent labeling of RNA by enzymatic transglycosylation. *Journal of the American Chemical Society* **137**, 12756–12759 (2015).
