# Supplementary material for: Highly efficient expression of DNA-peptide conjugates in growth-arrested cells
Source: Nat Commun. 2026 Jan 7;17:1422. doi: 10.1038/s41467-025-68167-5 (PMC12881617; doi:10.1038/s41467-025-68167-5)
Supplement: Supplementary file 3 — Description of Additional Supplementary Files [file 41467_2025_68167_MOESM3_ESM.pdf]

Description of Additional Supplementary Files

Supplementary Data 1: DNA coding sequences

a. pcDNA3.1 (+) eGFP coding sequence
b. miniGFP1 gene block coding sequence
c. ABE8e-P2A-eGFP coding sequence
d. Factor IX gene block coding sequence
